# Identification of stress resilience module by weighted gene co-expression network analysis in *Fkbp5*-deficient mice

**DOI:** 10.1186/s13041-019-0521-9

**Published:** 2019-11-27

**Authors:** Joonhong Kwon, Yeong Jae Kim, Koeul Choi, Sihwan Seol, Hyo Jung Kang

**Affiliations:** Department of Life Science, Chung-Ang University, 84 Heukseok-ro, Dongjak-gu, Seoul, 06974 South Korea

**Keywords:** FKBP5, Chronic stress, Resilience, RNAseq, WGCNA

## Abstract

*FKBP5* encodes the FK506 binding protein 5, a glucocorticoid receptor (GR) binding protein known to play an important role in the physiological stress response. However, results from previous studies examining the association between common variants of *FKBP5* and stress have been inconsistent. To investigate whether the loss of *FKBP5* affects the stress response, we examined the behavior of mice following the induction of chronic restraint stress between homozygous wild-type and *Fkbp5* knock-out mice. After 21 days of exposure to restraint stress, WT mice showed anhedonia, a core symptom of depression, which could be measured by a sucrose preference test. However, *Fkbp5*-deficient mice did not exhibit significant depressive-like behavior compared to the WT after exposure to chronic restraint stress. To investigate the molecular mechanism underlying stress resilience, we performed RNA sequencing analysis. The differentially expressed gene (DEG) analysis showed that chronic stress induced changes in various biological processes involved in cell-cell adhesion and inflammatory response. Weighted gene co-expression network analysis identified 60 characteristic modules that correlated with stress or the *FKBP5* genotype. Among them, M55 showed a gene expression pattern consistent with behavioral changes after stress exposure, and the gene ontology analysis revealed that this was involved in nervous system development, gland morphogenesis, and inflammatory response. These results suggest that *FKBP5* may be a crucial factor for the stress response, and that transcriptomic data can provide insight into stress-related pathophysiology.

## Main text

**Fig. 1:**
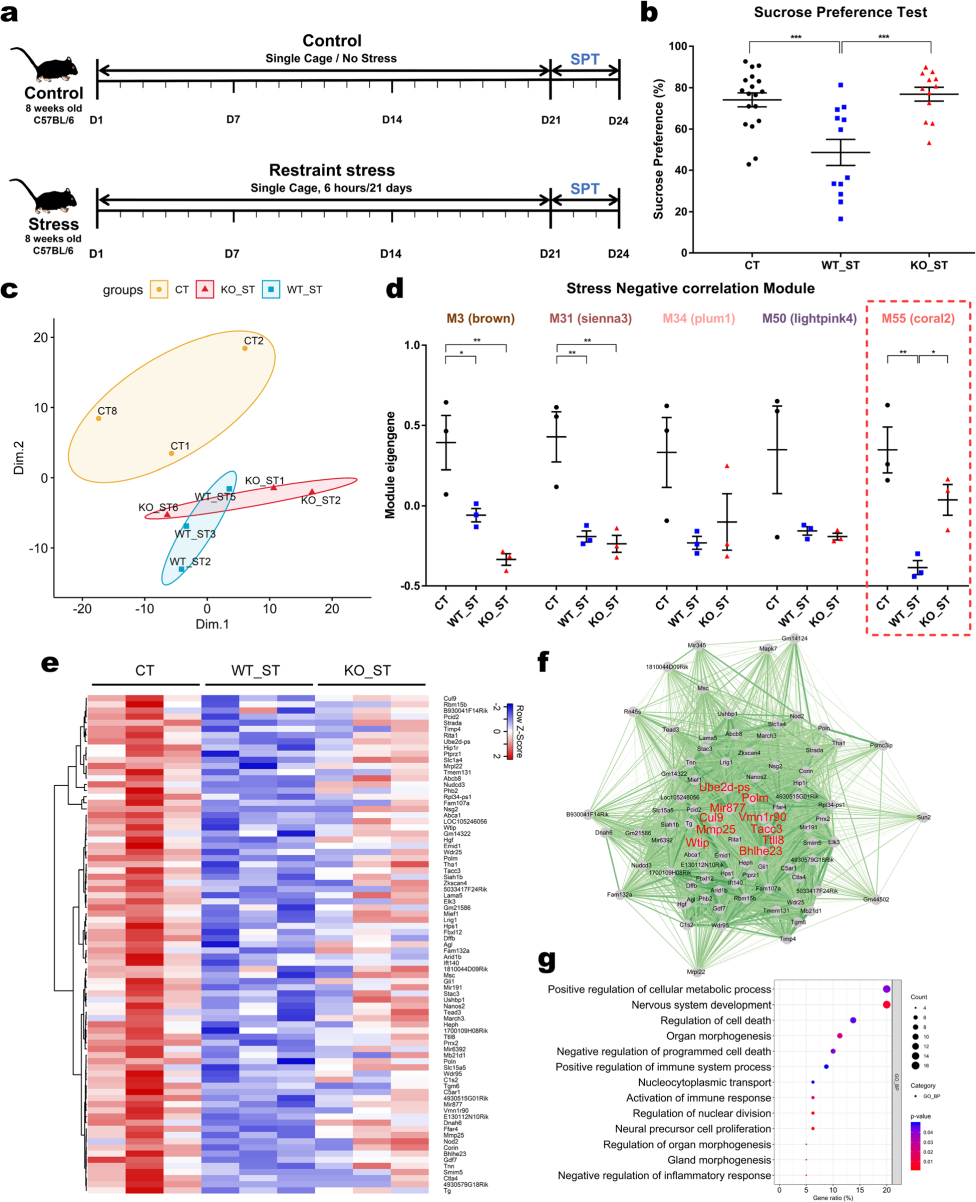
Transcriptome analyses of the mPFC in homozygous wild-type (WT) and *Fkbp5* knock-out (KO) mice following chronic stress. **a** Schematic timeline of the induction of chronic restraint stress. **b** Effect of CRS on sucrose preference. Data were combined with the 1st and 2nd sucrose preference test (SPT) results. Control mice (CT) *n* = 9; CRS-exposed WT mice (WT_ST) *n* = 6; CRS-exposed *Fkbp5* KO mice (KO_ST) *n* = 6. One-way ANOVA (F[2,39] = 11.67, *p* = 0.0001); Fisher’s LSD: ***p < 0.001. **c** Multidimensional scaling (MDS) plot for transcriptomes of individual samples of CT (yellow), WT_ST (blue) or KO_ST (red). **d** The interleaved scatter plots of modules which have a significant negative correlation with stress. Data represent mean ± SEM. One-way ANOVA; Fisher’s LSD: *p ≤ 0.05, **p < 0.01. **e** A heatmap showing the expression of M55 genes in the mPFC of CT (left), WT_ST (middle) and KO_ST (right). **f** A network plot of M55 genes and their intramodular connections. The ten hub genes (the top ten genes with highest intramodular connectivity; *Cul9, Polm, Ttll8, Vmn1r90, Tacc3, Mir877, Mmp25, Bhlhe23, Wtip* and *Ube2d-ps*) are shown in red. Note their central position in the network, suggesting high intramodular connectivity. **g** Enrichment dot plot for Gene Ontology (GO) analysis of M55 genes. The 13 GO terms with the lowest *p*-value each annotation cluster are plotted in order of gene ratio. The dot size represents the number of genes associated with a specific term. The dot color represents the adjusted p-value

Depression is one of the most common mental disorders affecting people of all ages, and can arise from a variety of causes, including genetic susceptibility, endocrine dysregulation, and stresses in life [1]. When exposed to acute and temporary stress, while the body can protect itself from stress, chronic stress can disturb the function of the brain system. Chronic accumulation of stress leads to abnormal and excessive cortisol secretion in the hypothalamic-pituitary-adrenal axis, which affects a variety of physical activities, including brain function, leading to mental disorders such as depression or post-traumatic stress disorder (PTSD) [2]. Proper regulation through negative feedback of glucocorticoid receptors (GR) is important for the stress response, and long-term or excessive activation of this system is associated with the development of depression or anxiety disorders [3]. The FK506-binding protein 51 (FKBP5) is a co-chaperone of Hsp90 in the GR molecular complex and is a key modulator of GR sensitivity [4, 5]. Although FKBP5 is an important factor that is responsible for coping behavior as well as neuroendocrine responses [6], results from previous studies investigating the association between *Fkbp5* gene variants and stress remain controversial. In order to investigate whether genetic *FKBP5* variants affect behavior in response to chronic restraint stress exposure, we examined the behavior of mice following the induction of chronic restraint stress in homozygous wild-type (WT) and knock-out (KO) mice. After 21 days of exposure to restraint stress, while WT mice showed anhedonia in the sucrose preference test, *Fkbp5*-deficient mice did not exhibit significant depressive-like behavior compared to the WT (Fig. 1)a and b.

Recent studies performed in humans and rodents have suggested that long-term stress and pathological anxiety leads to structural degeneration and functional alteration of the frontal cortex, and increases the risk of mental disorders [7, 8]. In addition, it has been suggested that the medial prefrontal cortex (mPFC), a region controlling higher brain function including cognition and emotion, is a primary target of stress hormones [9, 10]. However, little is known about the molecular mechanisms in the mPFC involved in stress-associated psychiatric disorders. Transcriptome profiling has helped provide an unbiased insight into the pathophysiological mechanism underlying complicated brain disorders [11]. Therefore, using RNA sequencing (RNAseq) analysis, we investigated the dynamic transcriptomic changes that occur after stress in the mPFC of *Fkbp5*-deficient mice.

Multidimensional scaling analysis showed a clear separation between the stressed and the non-stressed control (CT) mice. There was also a slight overlap between the CRS-exposed WT mice (WT_ST) and CRS-exposed *Fkbp5* KO mice (KO_ST) groups, which were clustered according to their genotype (Fig. 1)c. To identify the genotypes and genes affected by stress, we analyzed differentially expressed genes (DEG) between each group (Additional file 1). Of the 24,532 mRNA genes profiled, 224 (0.91%; CT vs WT_ST), 258 (1.05%; CT vs KO_ST), and 135 (0.55%; WT_ST vs KO_ST) genes were dysregulated in the mPFC following chronic restraint stress, and the percentage of DEG induced by stress was higher than the percentage of DEG by genotype (Additional file 2: Figure S1, Additional file 3: Table S1-S3). Gene ontology (GO) enrichment analysis of these expression profiles showed functional categories that were potentially dysregulated, including lipid metabolic process (*Benjamini adjusted p = 9.63 × 10*^*− 4*^), regulation of immune response (*Benjamini adjusted p = 7.24 × 10*^*− 4*^), cell adhesion (*Benjamini adjusted p = 1.52 × 10*^*− 3*^), regulation of cell differentiation (*Benjamini adjusted p = 1.17 × 10*^*− 3*^), and neurogenesis (*Benjamini adjusted p = 7.66 × 10*^*− 2*^) (Additional file 4: Table S4-S6).

To compare the differences in expression observed in the multidimensional data set with the pattern of stress-response behavior, we performed a weighted gene co-expression network analysis (WGCNA) [12]. Through WGCNA, we identified 60 modules of co-expressed genes following chronic restraint stress in both WT and KO homozygous genotypes (Additional file 5: Figure S2, Additional file 6: Table S7). Among the 60 modules, we identified characteristic modules which showed a significant correlation to the genotype (M3, M6, M25, M33, M39, M44 and M57) and to the stress exposure (M3, M10, M18, M21, M31, M34, M50 and M55) (Fig. 1, Additional file 7: Figure S3)d, a-c. Interestingly, one of the modules that negatively correlated with stress, M55, had a pattern similar to the stress resilience behavior of *Fkbp5*-deficient mice. This was down-regulated, consistent with depression-like behavior in the WT_ST group, and was restored in the KO_ST group (Fig. 1)d and e. GO enrichment analysis of M55 revealed the biological functions potentially involved, including gland morphogenesis (*p = 1.25 × 10*^*− 2*^), activation of immune response (*p = 2.25 × 10*^*− 2*^), and nervous system development (*p = 7.65 × 10*^*− 3*^) (Fig. 1, Additional file 8: Table S8)f and g. Hub genes, with the highest degree of connectivity within a module of the M55 include *Mmp25*. This gene has been functionally implicated in the regulation of immune response through NF-B signaling [13] and has been linked to neuropsychiatric disorders including PTSD [14].

In this study, we compared brain transcriptome altered by chronic stress in the mPFC between *Fkbp5*-deficient and wild-type mice by RNAseq analysis. In addition to the DEG analysis, by employing WGCNA, we identified a distinct co-expression network module associated with stress resilience caused by *Fkbp5* knock-out, and characterized the biological processes affected by this module, leading to this unique behavior. Our systematic transcriptome analysis demonstrated that aberration in the development of the neuroendocrine system, and regulation of the immune response may underlie the stress resilient behavior observed in the *Fkbp5* deficient mice. This is the first study, to the best of our knowledge, to identify the stress resilience associated genes through gene co-expression network analysis in *Fkbp5* deficient mice. Altogether, we confirmed that FKBP5 may be an important component of the stress response, suggesting that identification of the module associated with the stress response can provide a treatment strategy and therapeutic target to attenuate the depressive symptoms caused by stress.

## Supplementary information

**Additional file 1.** Materials and Methods.

**Additional file 2: Figure S1.** Heatmap representing the expression profiles of the DEGs in the three groups of CT, WT_ST, and KO_ST.

**Additional file 3: Table S1.** List of DEG between WT ST and CT. **Table S2.** List of DEG between KO ST and CT. **Table S3.** List of DEG between KO ST and WT ST.

**Additional file 4: Table S4.** GO analysis for DEGs between WT ST and CT. **Table S5.** GO analysis for DEGs between KO ST and CT. **Table S6.** GO analysis for DEGs between KO ST and WT ST.

**Additional file 5: Figure S2.** Heatmap of the correlation of WGCNA modules with traits. The correlation between each module eigengene and sample trait was calculated. Values in the figure indicate the correlation coefficient between modules and traits. Values in brackets are the *p*-values for the association test.

**Additional file 6: Table S7.** List of genes within each WGCNA module (kME > 0.7).

**Additional file 7: Figure S3.** The interleaved scatter plots of module which have a significant correlation with genotype and stress **(a-b)** Modules with positively (a) and negatively (b) correlated to the genotype. **(c)** Modules with positively correlated to the stress. Data represent mean ± SEM. One-way ANOVA; Fisher’s LSD: *p ≤ 0.05, **p < 0.01, ***p < 0.001.

**Additional file 8: Table S8.** GO analysis for genes in M55.

## Data Availability

The datasets supporting the conclusions of this article are included in this published article and its additional files. RNAseq raw data reported in this paper are submitted to the GEO repository under the accession number GSE138240.

## Abbreviations

bh.pval: Benjamini-Hochberg adjusted *P*-value
CRS: Chronic restraint stress
CT: Control
DEG: Differentially expressed genes
GO: Gene ontology
KO: Knock-out
KO_ST: CRS-exposed *Fkbp5* KO mice
mPFC: Medial prefrontal cortex
PTSD: Post-traumatic stress disorder
RNAseq: RNA sequencing
WGCNA: Weighted gene co-expression network analysis
WT: Wild-type
WT_ST: CRS-exposed WT mice
