# Supplementary material for: Identification of stress resilience module by weighted gene co-expression network analysis in Fkbp5-deficient mice
Source: Mol Brain. 2019 Nov 27;12:99. doi: 10.1186/s13041-019-0521-9 (PMC6882145; doi:10.1186/s13041-019-0521-9)
Supplement: Supplementary file 1 — Additional file 1. Materials and Methods. [file 13041_2019_521_MOESM1_ESM.docx]

**Additional File 1**

Identification of stress resilience module by weighted gene co-expression network analysis in Fkbp5 deficient mice

Joonhong Kwon^1^, YeongJae Kim^1^, Koeul Choi^1^, Sihwan Seol^1^, and Hyo Jung Kang*^1^

^1^Department of Life Science, Chung-Ang University, Seoul 06974, South Korea

**Materials and Methods**

**Animal**

Eight-week-old male C57BL/6J mice maintained in the vivarium at Chung-Ang University were used for the experiments. All mice were housed in a single cage, provided with unlimited access to food and water, maintained at a 12-hour light-dark cycle, and tested during the light phase. The mice were sacrificed by cervical dislocation. Immediately after decapitation, the brains were rapidly removed, rinsed with ice-cold phosphate-buffered saline, and placed into a brain matrix (Cat # SA-2175, Roboz Surgical Instruments, Gaithersburg, MD). Coronal brain slices of 1.0 mm thick were collected and transferred to the RNA stabilization solution (RNA*later*, Cat # AM7021, ThermoFisher Scientific, Waltham, MA). The medial prefrontal cortex (mPFC) region was dissected according to the atlas (Paxinos and Franklin, 4^th^ Edition). Strains of *Fkbp5* KO mice (JAX stock #017989) were obtained from The Jackson Laboratory (Bar Harbor, Maine, USA), and the *Fkbp5* KO mice used for this study were backcrossed with C57BL/6J for ten generations. All mice used in the experiments were obtained by heterozygous mating (*Fkbp5*^+/-^ x *Fkbp5*^+/-^) according to the Chung-Ang University Research Requirements. All procedures were approved by the Committee on the Institutional Animal Care and Use of Chung-Ang University.

**Chronic Restraint Stress (CRS)**

Mice of the stressed group were horizontally immobilized for 6 hours per day in a translucent latex glove for 3 weeks in their home cages. The thumb area of a latex glove made of translucent material was cut with scissors, and the mouse tail was taken out through the thumb hole of the latex glove and wrapped around the latex glove to induce chronic stress. Non-restraint mice (CT; n=9) remained in their home cages without the CRS procedure. Both CT and CRS mice (WT ST; n=6, KO ST; n=6) did not have access to food and water during the period of CRS exposure.

**Behavioral Test (Sucrose Preference Test)**

The habituation process was performed to prevent water phobia against 1% sucrose water 24 hours before obtaining sucrose preference test (SPT) measurements. After the habituation process, water deprivation was performed for 24 hours. Mice were presented with two bottles: one filled with tap water and the other with 1% sucrose solution (200 ml each). The 24-hour consumption of water and sucrose solution was calculated by subtracting the weight of the bottles. Sucrose preference (%) was calculated as consumption of sucrose solution divided by total fluid consumption (i.e., water plus sucrose), multiplied by 100.

**Total RNA extraction**

Total RNA extraction was performed using miRNeasy mini kit (Qiagen, 217004). Mice mPFC tissue was homogenized in QIAzol lysis reagent. The samples were combined with chloroform and centrifuged for 15 min at 12,000 *g* and 4^o^C. The supernatant was combined with 100% ethanol and applied to spin column. Before elution, DNase I treatment (Qiagen, 79254) was carried out directly on the spin column. Subsequent processes were performed according to the manufacturer's instructions.

**RNA Sequencing**

Library preparations and sequencing were performed by Macrogen Inc. (Seoul, Korea). TruSeq Stranded mRNA LT Sample Prep Kit (Illumina, USA) was used to synthesize cDNA libraries according to the manufacturer’s instructions. HiSeq 4000 (Illumina, USA) was used for sequencing to generate 101-bp paired end reads. A quality control procedure, known as preprocessing, was performed to eliminate artifacts (low quality or contaminated DNA). Once the sequence of the reads was determined, they were aligned to the reference genome (GRCm38) using the HISAT2 (Hierarchical Indexing for Spliced Alignment of Transcripts) program which handles spliced read mapping through the Bowtie2 aligner, to generate aligned reads. Based on this data, we proceeded with transcript assembly using the StringTie program. Expression profile was extracted from Fragments Per Kilobase of transcript per Million mapped reads (FPKM) value by expression quantification obtained from the transcript quantification of each sample. Differentially expressed genes (DEGs) were identified after quantile normalization of the log2 (FPKM+1) values followed by student's *T*-test (p < 0.05). The P-values were corrected by using the p.adjust function (method = “Benjamini–Hochberg”) in R. Genes with a |fold change| value greater than 1.2, and raw.p value less than 0.05 were considered as differentially expressed. To exclude false positive genes, genes with low expression levels (baseMean <30) were excluded from the list of DEGs.

**Gene Functional Enrichment Analysis**

Gene ontology (GO) and Kyoto Encyclopedia of Genes and Genomes (KEGG) pathway enrichment analysis was performed to explore the functional roles of DEGs from mRNA-seq data and genes in selected modules using the Database for Annotation, Visualization and Integrated Discovery version 6.8 (DAVID; http://david.ncifcrf.gov/). The enrichment results were visualized using the ‘ggplot2’ package of the R program. Statistically significant categories were selected (p-value<0.05).

**Weighted Gene Co-expression Network Analysis (WGCNA)**

Signed hybrid co-expression networks in mPFC were constructed using the weighted gene co-expression network analysis (WGCNA) package in R using RNA-seq data [12]. For all genes included, a pairwise correlation matrix was computed, and an adjacency matrix was calculated by raising the correlation matrix to a power of 8 for all networks, according to a scale-free topology criterion [12]. For each pair of genes, a robust measurement of network interconnectedness (topological overlap measure) was calculated based on the adjacency matrix. The topological overlap-based dissimilarity was then used as input for average linkage hierarchical clustering. Modules were generated by hybrid dynamic tree-cutting. To obtain co-expression patterns, we set the minimum module size to 30 genes, deepSplit to 2, and the minimum height for merging modules to 0.25. Each module was summarized by an eigengene, which was the first principal component of the scaled module expression. To obtain cleaner modules, we defined the module membership measure (also known as module eigengene based connectivity kME) as the correlation between gene expression values and the module eigengene. Genes with kME>0.7 were cut-off. The module membership was also used to rank genes in the module. Top ten genes in the rank were considered as hub genes of the module.

**References**

1. Langfelder P, Horvath S. WGCNA: an R package for weighted correlation network analysis. BMC Bioinformatics. 2008;9:559
