# Supplementary material for: Identification of stress resilience module by weighted gene co-expression network analysis in Fkbp5-deficient mice
Source: Mol Brain. 2019 Nov 27;12:99. doi: 10.1186/s13041-019-0521-9 (PMC6882145; doi:10.1186/s13041-019-0521-9)

**Figure S1.** Heatmap representing the expression profiles of the DEGs in the three groups of CT, WT_ST, and KO_ST


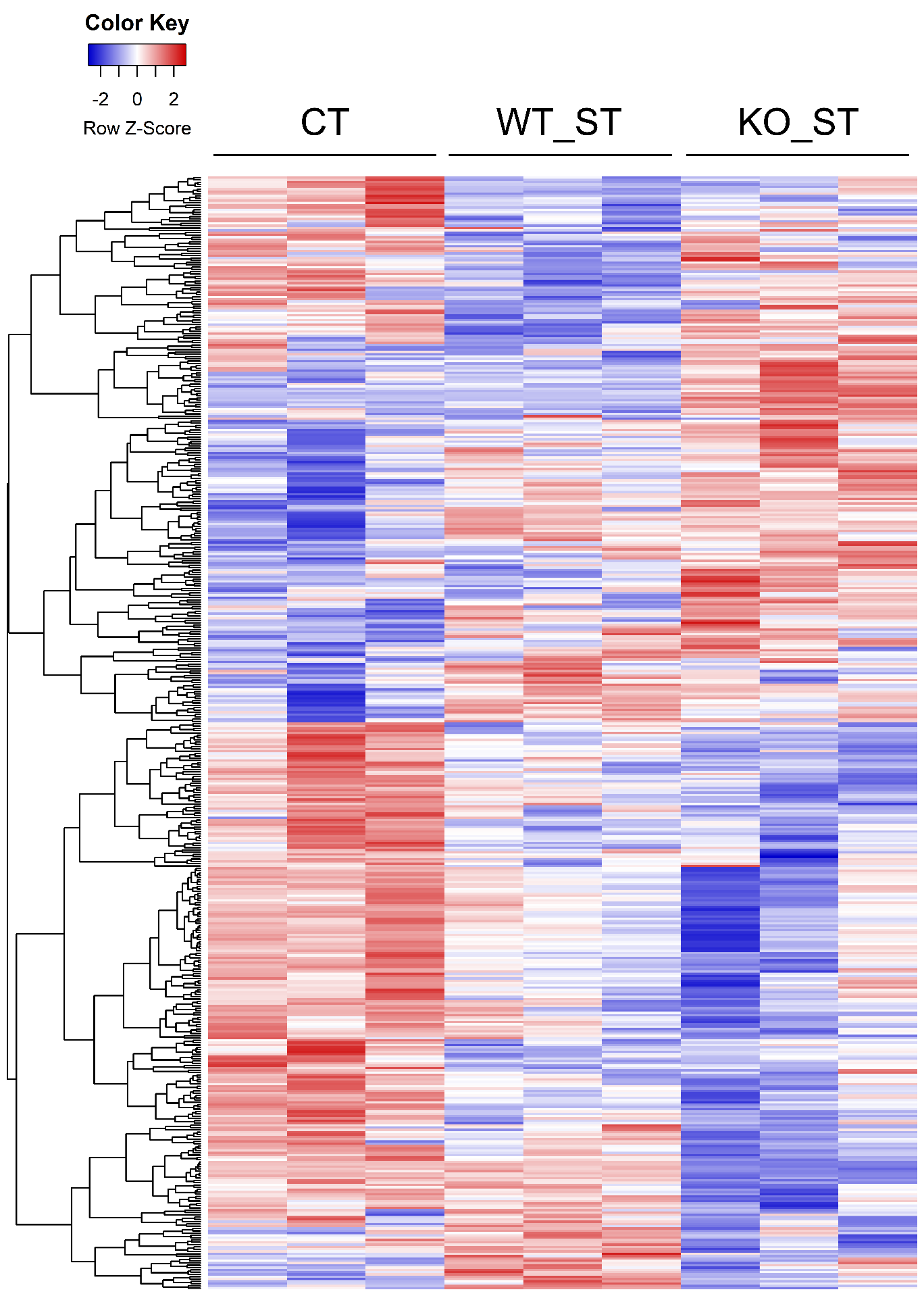

Supplement: Supplementary file 2 — Additional file 2: Figure S1. Heatmap representing the expression profiles of the DEGs in the three groups of CT, WT_ST, and KO_ST. [file 13041_2019_521_MOESM2_ESM.docx]
