# Supplementary material for: Identification of stress resilience module by weighted gene co-expression network analysis in Fkbp5-deficient mice
Source: Mol Brain. 2019 Nov 27;12:99. doi: 10.1186/s13041-019-0521-9 (PMC6882145; doi:10.1186/s13041-019-0521-9)

**Figure S2.** Heatmap of the correlation of WGCNA modules with traits. The correlation between each module eigengene and sample trait was calculated. Values in the figure indicate the correlation coefficient between modules and traits. Values in brackets are the p-values for the association test.


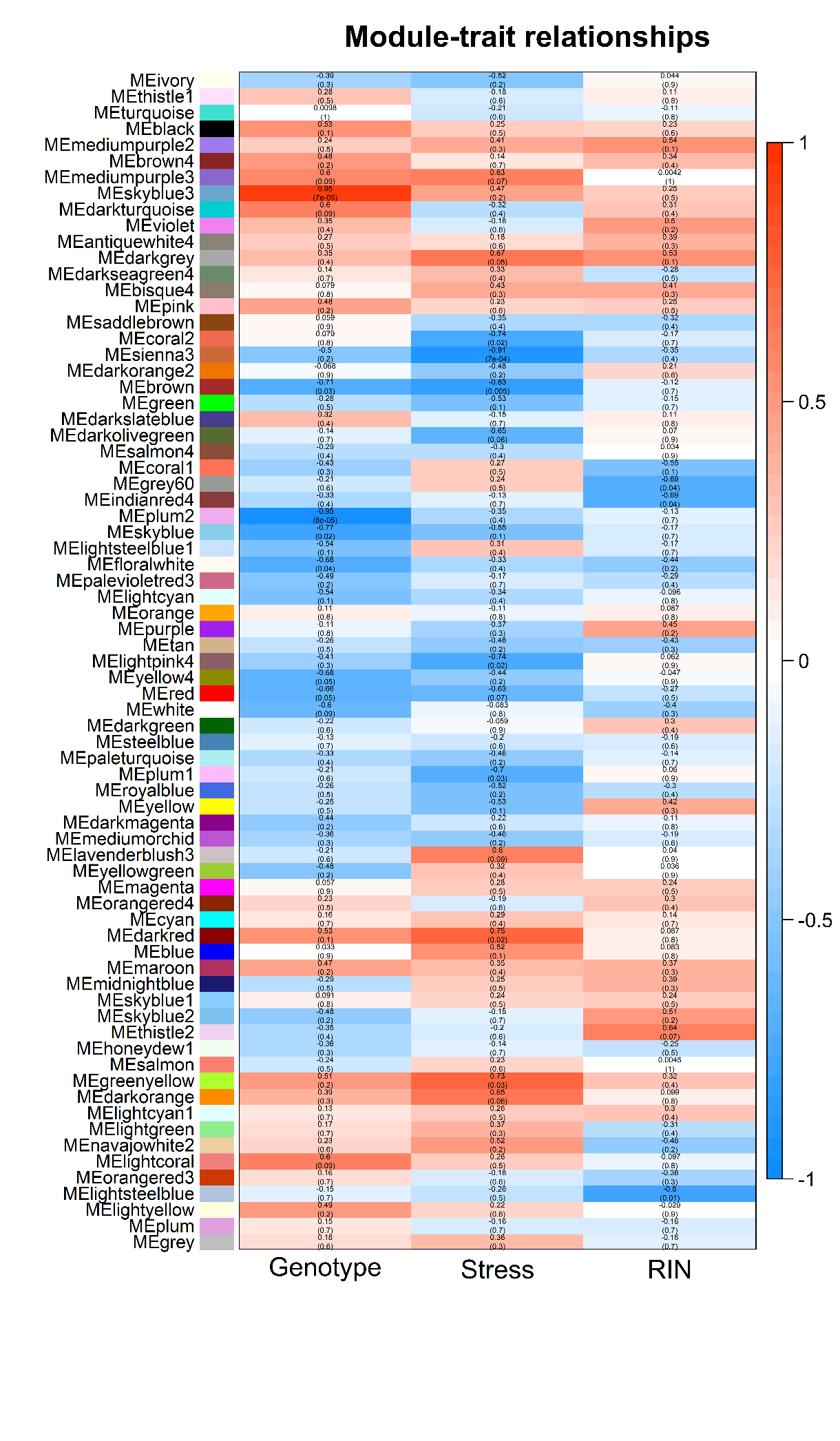

Supplement: Supplementary file 5 — Additional file 5: Figure S2. Heatmap of the correlation of WGCNA modules with traits. The correlation between each module eigengene and sample trait was calculated. Values in the figure indicate the correlation coefficient between modules and traits. Values in brackets are the p-values for the association test. [file 13041_2019_521_MOESM5_ESM.docx]
