# Supplementary material for: Identification of stress resilience module by weighted gene co-expression network analysis in Fkbp5-deficient mice
Source: Mol Brain. 2019 Nov 27;12:99. doi: 10.1186/s13041-019-0521-9 (PMC6882145; doi:10.1186/s13041-019-0521-9)

**Figure S3.** The interleaved scatter plots of module which have a significant correlation with genotype and stress **(a-b)** Modules with positively (a) and negatively (b) correlated to the genotype. **(c)** Modules with positively correlated to the stress. Data represent mean±SEM. One-way ANOVA; Fisher’s LSD: *p≤0.05, **p<0.01, ***p<0.001.


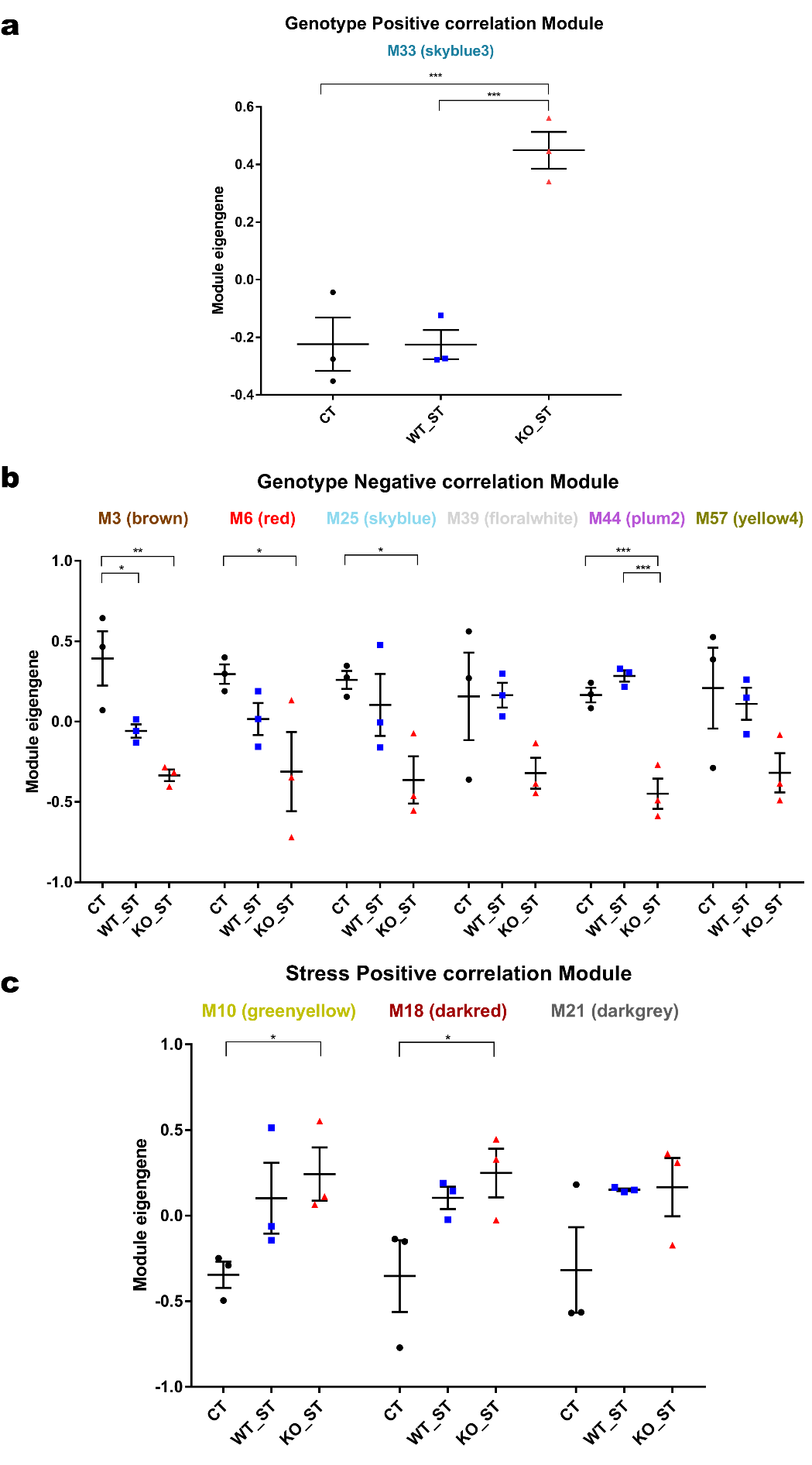

Supplement: Supplementary file 7 — Additional file 7: Figure S3. The interleaved scatter plots of module which have a significant correlation with genotype and stress (a-b) Modules with positively (a) and negatively (b) correlated to the genotype. (c) Modules with positively correlated to the stress. Data represent mean ± SEM. One-way ANOVA; Fisher’s LSD: *p ≤ 0.05, **p < 0.01, ***p < 0.001. [file 13041_2019_521_MOESM7_ESM.docx]
